# Bis(2-amino-3*H*-benzothia­zolium) bis­(7-oxabicyclo­[2.2.1]heptane-2,3-dicarboxyl­ato)cobaltate(II) hexa­hydrate

**DOI:** 10.1107/S1600536810020921

**Published:** 2010-06-09

**Authors:** Na Wang, Qiu-Yue Lin, Jie Feng, Shi-Kun Li, Jun-Jun Zhao

**Affiliations:** a Zhejiang Key Laboratory for Reactive Chemistry on Solid Surfaces, Institute of Physical Chemistry, Zhejiang Normal University, Jinhua, Zhejiang 321004, People’s Republic of China; b College of Chemistry and Life Science, Zhejiang Normal University, Jinhua 321004, Zhejiang, People’s Republic of China

## Abstract

In the crystal structure of the title salt, (C_7_H_7_N_2_S)_2_[Co(C_8_H_8_O_5_)_2_]·6H_2_O, the heterocyclic N atom of the 2-amino­benzothia­zole mol­ecule is protonated. The Co^II^ atom is situated on an inversion centre and exhibits a slightly distorted octa­hedral CoO_6_ coordination defined by the bridging O atoms of the bicyclo­heptane unit and four carboxyl­ate O atoms of two symmetry-related and fully deprotonated ligands. The crystal packing is stabilized by N—H⋯O hydrogen bonds between the cations and anions and by O—H⋯O hydrogen bonds including the crystal water mol­ecules.

## Related literature

7-Oxabicyclo­[2.2.1]heptane-2,3-dicarb­oxy­lic anhydride (nor­cantharidin) is a lower toxicity anti­cancer drug, see: Shimi *et al.* (1982 ▶). For the importance of cobalt in biological systems, see: Jiao *et al.* (2005 ▶). For the isotypic structure of the Mn analogue, see: Wang *et al.* (2010 ▶). For related cobalt complexes, see: Wang *et al.* (1988 ▶, 2009 ▶).
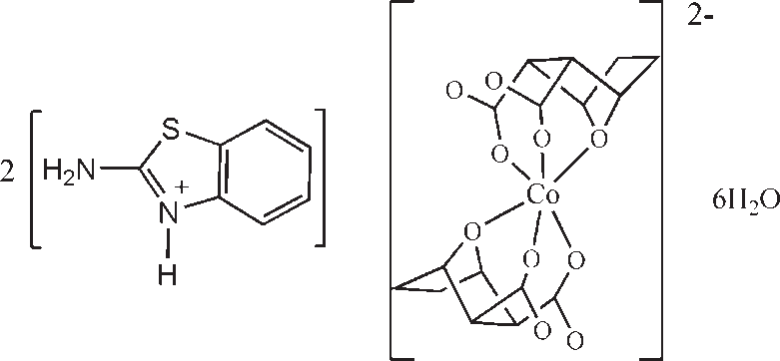


## Experimental

### 

#### Crystal data

(C_7_H_7_N_2_S)_2_[Co(C_8_H_8_O_5_)_2_]·6H_2_O
*M* *_r_* = 837.73
Triclinic, 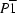

*a* = 6.6924 (4) Å
*b* = 10.1294 (5) Å
*c* = 13.1860 (7) Å
α = 90.094 (4)°
β = 91.112 (4)°
γ = 99.314 (4)°
*V* = 881.92 (8) Å^3^
*Z* = 1
Mo *K*α radiation
μ = 0.69 mm^−1^
*T* = 296 K
0.19 × 0.16 × 0.07 mm

#### Data collection

Bruker APEXII area-detector diffractometer
Absorption correction: multi-scan *SADABS* (Sheldrick, 1996 ▶) *T*_min_ = 0.876, *T*_max_ = 0.953
13051 measured reflections
3999 independent reflections
2460 reflections with *I* > 2σ(*I*)
*R*_int_ = 0.051

#### Refinement

*R*[*F* ^2^ > 2σ(*F* ^2^)] = 0.052
*wR*(*F* ^2^) = 0.130
*S* = 1.03
3999 reflections
262 parameters
10 restraints
H atoms treated by a mixture of independent and constrained refinement
Δρ_max_ = 0.47 e Å^−3^
Δρ_min_ = −0.44 e Å^−3^

### 

Data collection: *APEX2* (Bruker, 2006 ▶); cell refinement: *SAINT* (Bruker, 2006 ▶); data reduction: *SAINT*; program(s) used to solve structure: *SHELXS97* (Sheldrick, 2008 ▶); program(s) used to refine structure: *SHELXL97* (Sheldrick, 2008 ▶); molecular graphics: *SHELXTL* (Sheldrick, 2008 ▶); software used to prepare material for publication: *SHELXL97*.

## Supplementary Material

Crystal structure: contains datablocks I, global. DOI: 10.1107/S1600536810020921/wm2351sup1.cif

Structure factors: contains datablocks I. DOI: 10.1107/S1600536810020921/wm2351Isup2.hkl

Additional supplementary materials:  crystallographic information; 3D view; checkCIF report

## Figures and Tables

**Table 1:** Selected bond lengths (Å)

Co1—O4	2.033 (2)
Co1—O2	2.110 (2)
Co1—O5	2.160 (2)

**Table 2:** Hydrogen-bond geometry (Å, °)

*D*—H⋯*A*	*D*—H	H⋯*A*	*D*⋯*A*	*D*—H⋯*A*
N1—H1*N*⋯O1^i^	0.84 (2)	1.85 (2)	2.675 (3)	169 (3)
N2—H2*C*⋯O2^i^	0.86	2.00	2.851 (3)	173
N2—H2*D*⋯O2*W*^ii^	0.86	2.01	2.828 (4)	160
O1*W*—H1*WA*⋯O3*W*^ii^	0.82 (2)	2.21 (2)	3.030 (4)	176 (4)
O1*W*—H1*WB*⋯O3*W*^iii^	0.84 (4)	1.94 (2)	2.769 (4)	171 (5)
O2*W*—H2*WA*⋯O3	0.85 (2)	1.85 (2)	2.686 (3)	167 (4)
O2*W*—H2*WB*⋯O1*W*	0.83 (2)	1.95 (2)	2.772 (4)	171 (4)
O3*W*—H3*WA*⋯O1	0.84 (2)	2.01 (2)	2.815 (3)	160 (4)
O3*W*—H3*WB*⋯O2*W*	0.83 (4)	1.96 (4)	2.790 (4)	178 (4)

Symmetry codes: (i) 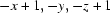; (ii) 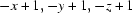; (iii) 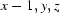.

**Fig. 1.**
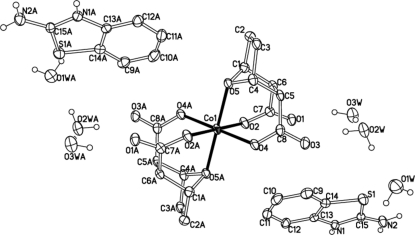
A view of the molecular units of the title salt showing the atom-labelling scheme with displacement ellipsoids drawn at the 30% probability. Symmetry code: A (-x+2, -y, -z).

## supplementary crystallographic information

### Comment

7-oxabicyclo[2,2,1] heptane-2,3-dicarboxylic anhydride (norcantharidin) derived from cantharidin is a lower toxicity anticancer drug (Shimi *et al.*, 1982). Cobalt was recognized as an essential metal element widely distributed in biological systems such as cells and body (Jiao *et al.*, 2005). Several related cobalt complexes with the same ligand  (Wang *et al.*, 1988) and with the ligand and with imidazole (Wang *et al.*, 2009) have been reported.

In the title complex, (C_7_H_7_N_2_S)^+^_2_[Co(C_8_H_8_O_5_)_2_]^2-^(H_2_O)_6_, the Co^II^ ion is located on a crystallographic centre of inversion. Two bridging oxygen atoms of the bicycloheptane units and four carboxylate oxygen atoms give rise to a slightly distorted octahedral coordination environment around the Co^II^ atom. The bond angles O2—Co1—O2^i^, O4—Co1—O4^i^ and O5—Co1—O5^i^ (i: -x+1, -y, -z.) are 180°, while the bond angles O4—Co1—O2 and O2—Co1—O4^i^ open up slightly from 87.71 (9)° to 92.29 (9)°, resulting in a slight distortion from the ideal octahedral geometry. The crystal packing is stabilized by N—H···O hydrogen bonds between the cations and anions and by O—H···O hydrogen bonds including the crystal water molecules.

The crystal structure of (C_7_H_7_N_2_S)^+^_2_[Co(C_8_H_8_O_5_)_2_]^2-^(H_2_O)_6_ is isotypic with that of the Mn analogue (Wang *et al.*, 2010) where slightly longer metal—oxygen bonds are observed.

### Experimental

Norcantharidin, cobalt acetate and 2-aminobenzothiazole were dissolved in 15 mL distilled water. The mixture was sealed in a 25 mL Teflon-lined stainless vessel and heated at 443 K for 3 d, then cooled slowly to room temperature. Pink crystals suitable for X-ray diffraction were obtained.

### Refinement

The H atoms bonded to C and N atoms were positioned geometrically and refined using a riding model [aromatic C—H = 0.93 Å, aliphatic C—H = 0.97–0.98 Å and N—H = 0.86 Å and *U*_iso_(H)=1.2U_eq_(parent atom)]. The H atoms of the water molecule were located in a difference Fourier maps and refined with O—H distance restraints of 0.85 (2) and *U*_iso_(H) = 1.5U_eq_(O).

### Crystal data

**Table:**

(C_7_H_7_N_2_S)_2_[Co(C_8_H_8_O_5_)_2_]·6H_2_O	*Z* = 1
*M**_r_* = 837.73	*F*(000) = 437
Triclinic, *P*1	*D*_x_ = 1.577 Mg m^−^^3^
Hall symbol: -P 1	Mo *K*α radiation, λ = 0.71073 Å
*a* = 6.6924 (4) Å	Cell parameters from 2197 reflections
*b* = 10.1294 (5) Å	θ = 1.5–27.6°
*c* = 13.1860 (7) Å	µ = 0.69 mm^−^^1^
α = 90.094 (4)°	*T* = 296 K
β = 91.112 (4)°	Block, pink
γ = 99.314 (4)°	0.19 × 0.16 × 0.07 mm
*V* = 881.92 (8) Å^3^	

### Data collection

**Table:**

Bruker APEXII area-detector diffractometer	3999 independent reflections
Radiation source: fine-focus sealed tube	2460 reflections with *I* > 2σ(*I*)
graphite	*R*_int_ = 0.051
ω scans	θ_max_ = 27.6°, θ_min_ = 1.5°
Absorption correction: multi-scan *SADABS* (Sheldrick, 1996)	*h* = −8→8
*T*_min_ = 0.876, *T*_max_ = 0.953	*k* = −11→13
13051 measured reflections	*l* = −17→17

### Refinement

**Table:**

Refinement on *F*^2^	Primary atom site location: structure-invariant direct methods
Least-squares matrix: full	Secondary atom site location: difference Fourier map
*R*[*F*^2^ > 2σ(*F*^2^)] = 0.052	Hydrogen site location: inferred from neighbouring sites
*wR*(*F*^2^) = 0.130	H atoms treated by a mixture of independent and constrained refinement
*S* = 1.03	*w* = 1/[σ^2^(*F*_o_^2^) + (0.0599*P*)^2^] where *P* = (*F*_o_^2^ + 2*F*_c_^2^)/3
3999 reflections	(Δ/σ)_max_ < 0.001
262 parameters	Δρ_max_ = 0.47 e Å^−^^3^
10 restraints	Δρ_min_ = −0.44 e Å^−^^3^

### Special details

**Table:**

Geometry. All e.s.d.'s (except the e.s.d. in the dihedral angle between two l.s. planes) are estimated using the full covariance matrix. The cell e.s.d.'s are taken into account individually in the estimation of e.s.d.'s in distances, angles and torsion angles; correlations between e.s.d.'s in cell parameters are only used when they are defined by crystal symmetry. An approximate (isotropic) treatment of cell e.s.d.'s is used for estimating e.s.d.'s involving l.s. planes.
Refinement. Refinement of *F*^2^ against ALL reflections. The weighted *R*-factor *wR* and goodness of fit *S* are based on *F*^2^, conventional *R*-factors *R* are based on *F*, with *F* set to zero for negative *F*^2^. The threshold expression of *F*^2^ > σ(*F*^2^) is used only for calculating *R*-factors(gt) *etc*. and is not relevant to the choice of reflections for refinement. *R*-factors based on *F*^2^ are statistically about twice as large as those based on *F*, and *R*- factors based on ALL data will be even larger.

### Fractional atomic coordinates and isotropic or equivalent isotropic displacement parameters (Å^2^)

**Table:**

	*x*	*y*	*z*	*U*_iso_*/*U*_eq_	
Co1	0.5000	0.0000	0.0000	0.0303 (2)	
S1	0.32716 (14)	0.26697 (8)	0.52780 (6)	0.0399 (2)	
N1	0.2766 (4)	0.0309 (2)	0.60086 (19)	0.0302 (6)	
H1N	0.277 (5)	−0.031 (3)	0.644 (2)	0.045*	
N2	0.3473 (4)	0.1974 (3)	0.7234 (2)	0.0406 (7)	
H2C	0.3394	0.1394	0.7713	0.049*	
H2D	0.3742	0.2814	0.7375	0.049*	
O1	0.7726 (3)	0.1597 (2)	0.25745 (15)	0.0384 (6)	
O1W	0.1942 (5)	0.5445 (3)	0.4012 (2)	0.0706 (8)	
H1WA	0.199 (7)	0.563 (5)	0.4621 (17)	0.106*	
H1WB	0.085 (5)	0.492 (4)	0.393 (3)	0.106*	
O2	0.6869 (3)	0.0130 (2)	0.13153 (15)	0.0376 (6)	
O2W	0.5190 (4)	0.5231 (2)	0.2784 (2)	0.0546 (7)	
H2WA	0.478 (6)	0.473 (4)	0.228 (2)	0.082*	
H2WB	0.416 (4)	0.522 (4)	0.313 (3)	0.082*	
O3	0.3619 (3)	0.3400 (2)	0.13885 (17)	0.0429 (6)	
O3W	0.8114 (5)	0.3962 (3)	0.3736 (2)	0.0608 (7)	
H3WA	0.828 (6)	0.334 (3)	0.334 (3)	0.091*	
H3WB	0.723 (6)	0.432 (4)	0.345 (3)	0.091*	
O4	0.3648 (3)	0.14697 (19)	0.06047 (16)	0.0369 (5)	
O5	0.7143 (3)	0.15513 (19)	−0.06851 (15)	0.0327 (5)	
C1	0.9035 (5)	0.1842 (3)	−0.0093 (2)	0.0334 (8)	
H1A	0.9718	0.1064	−0.0002	0.040*	
C2	1.0232 (5)	0.2949 (3)	−0.0715 (2)	0.0396 (8)	
H2A	1.0898	0.2587	−0.1274	0.047*	
H2B	1.1237	0.3514	−0.0299	0.047*	
C3	0.8570 (5)	0.3722 (3)	−0.1100 (2)	0.0397 (8)	
H3A	0.8797	0.4637	−0.0851	0.048*	
H3B	0.8483	0.3724	−0.1835	0.048*	
C4	0.6689 (5)	0.2912 (3)	−0.0646 (2)	0.0321 (7)	
H4A	0.5435	0.3016	−0.1011	0.039*	
C5	0.6600 (5)	0.3150 (3)	0.0495 (2)	0.0298 (7)	
H5A	0.6967	0.4107	0.0645	0.036*	
C6	0.8339 (5)	0.2379 (3)	0.0895 (2)	0.0301 (7)	
H6A	0.9443	0.3018	0.1201	0.036*	
C7	0.7589 (5)	0.1300 (3)	0.1659 (2)	0.0307 (7)	
C8	0.4489 (5)	0.2639 (3)	0.0876 (2)	0.0314 (7)	
C9	0.2570 (5)	0.1328 (4)	0.3395 (2)	0.0444 (9)	
H9A	0.2729	0.2130	0.3041	0.053*	
C10	0.2146 (5)	0.0118 (4)	0.2888 (3)	0.0497 (10)	
H10A	0.2011	0.0102	0.2185	0.060*	
C11	0.1922 (5)	−0.1068 (4)	0.3421 (3)	0.0466 (9)	
H11A	0.1645	−0.1874	0.3067	0.056*	
C12	0.2099 (5)	−0.1089 (3)	0.4467 (2)	0.0373 (8)	
H12A	0.1936	−0.1892	0.4820	0.045*	
C13	0.2522 (4)	0.0113 (3)	0.4966 (2)	0.0291 (7)	
C14	0.2750 (5)	0.1318 (3)	0.4437 (2)	0.0325 (7)	
C15	0.3185 (5)	0.1578 (3)	0.6290 (2)	0.0297 (7)	

### Atomic displacement parameters (Å^2^)

**Table:**

	*U*^11^	*U*^22^	*U*^33^	*U*^12^	*U*^13^	*U*^23^
Co1	0.0393 (4)	0.0196 (3)	0.0308 (4)	0.0010 (3)	−0.0019 (3)	−0.0021 (2)
S1	0.0525 (6)	0.0285 (5)	0.0373 (5)	0.0028 (4)	−0.0009 (4)	0.0063 (4)
N1	0.0372 (16)	0.0244 (15)	0.0291 (16)	0.0053 (12)	0.0018 (12)	0.0021 (11)
N2	0.058 (2)	0.0267 (15)	0.0354 (16)	0.0027 (13)	−0.0010 (13)	0.0014 (12)
O1	0.0575 (16)	0.0301 (12)	0.0265 (13)	0.0042 (11)	−0.0040 (10)	0.0005 (10)
O1W	0.068 (2)	0.074 (2)	0.0641 (19)	−0.0052 (16)	0.0018 (16)	−0.0069 (17)
O2	0.0504 (15)	0.0225 (12)	0.0367 (13)	−0.0029 (10)	−0.0079 (10)	0.0022 (9)
O2W	0.0644 (19)	0.0363 (15)	0.0614 (18)	0.0040 (13)	−0.0083 (14)	−0.0138 (13)
O3	0.0439 (15)	0.0339 (13)	0.0519 (15)	0.0093 (11)	0.0025 (11)	−0.0134 (11)
O3W	0.067 (2)	0.0509 (18)	0.0643 (19)	0.0122 (14)	−0.0146 (15)	−0.0112 (14)
O4	0.0377 (14)	0.0221 (12)	0.0493 (14)	−0.0002 (10)	0.0026 (10)	−0.0065 (10)
O5	0.0406 (13)	0.0233 (11)	0.0322 (12)	−0.0005 (10)	0.0010 (10)	−0.0025 (9)
C1	0.0352 (19)	0.0254 (17)	0.041 (2)	0.0085 (14)	0.0014 (15)	0.0004 (14)
C2	0.042 (2)	0.0328 (18)	0.042 (2)	0.0004 (16)	0.0076 (16)	0.0011 (15)
C3	0.059 (2)	0.0243 (17)	0.0333 (19)	−0.0023 (16)	0.0036 (16)	−0.0004 (14)
C4	0.042 (2)	0.0235 (16)	0.0312 (18)	0.0070 (14)	−0.0053 (14)	0.0016 (13)
C5	0.040 (2)	0.0162 (15)	0.0319 (18)	0.0022 (13)	−0.0031 (14)	−0.0021 (13)
C6	0.0338 (19)	0.0221 (16)	0.0325 (18)	−0.0005 (14)	−0.0042 (14)	−0.0009 (13)
C7	0.0327 (19)	0.0261 (17)	0.0334 (19)	0.0062 (14)	−0.0048 (14)	0.0034 (14)
C8	0.042 (2)	0.0246 (17)	0.0281 (17)	0.0072 (15)	−0.0037 (14)	0.0006 (14)
C9	0.048 (2)	0.053 (2)	0.032 (2)	0.0084 (18)	0.0041 (16)	0.0076 (17)
C10	0.047 (2)	0.075 (3)	0.0282 (19)	0.014 (2)	0.0015 (16)	−0.006 (2)
C11	0.039 (2)	0.053 (2)	0.047 (2)	0.0080 (18)	−0.0002 (17)	−0.0193 (19)
C12	0.037 (2)	0.0364 (19)	0.040 (2)	0.0114 (15)	0.0021 (15)	−0.0044 (16)
C13	0.0247 (18)	0.0316 (18)	0.0316 (18)	0.0061 (14)	0.0033 (13)	−0.0007 (14)
C14	0.0300 (18)	0.0328 (18)	0.0342 (19)	0.0038 (14)	0.0025 (14)	0.0029 (14)
C15	0.0334 (19)	0.0268 (17)	0.0283 (18)	0.0032 (14)	0.0024 (14)	−0.0019 (13)

### Geometric parameters (Å, °)

**Table:**

Co1—O4	2.033 (2)	C1—C2	1.520 (4)
Co1—O4^i^	2.033 (2)	C1—C6	1.521 (4)
Co1—O2	2.110 (2)	C1—H1A	0.9800
Co1—O2^i^	2.110 (2)	C2—C3	1.539 (5)
Co1—O5^i^	2.160 (2)	C2—H2A	0.9700
Co1—O5	2.160 (2)	C2—H2B	0.9700
S1—C15	1.730 (3)	C3—C4	1.521 (4)
S1—C14	1.747 (3)	C3—H3A	0.9700
N1—C15	1.322 (4)	C3—H3B	0.9700
N1—C13	1.392 (4)	C4—C5	1.527 (4)
N1—H1N	0.840 (17)	C4—H4A	0.9800
N2—C15	1.308 (4)	C5—C8	1.519 (4)
N2—H2C	0.8600	C5—C6	1.585 (4)
N2—H2D	0.8600	C5—H5A	0.9800
O1—C7	1.242 (3)	C6—C7	1.519 (4)
O1W—H1WA	0.823 (18)	C6—H6A	0.9800
O1W—H1WB	0.839 (19)	C9—C14	1.377 (4)
O2—C7	1.283 (3)	C9—C10	1.380 (5)
O2W—H2WA	0.852 (18)	C9—H9A	0.9300
O2W—H2WB	0.828 (18)	C10—C11	1.381 (5)
O3—C8	1.245 (4)	C10—H10A	0.9300
O3W—H3WA	0.842 (18)	C11—C12	1.383 (4)
O3W—H3WB	0.831 (18)	C11—H11A	0.9300
O4—C8	1.274 (3)	C12—C13	1.369 (4)
O5—C4	1.460 (3)	C12—H12A	0.9300
O5—C1	1.463 (4)	C13—C14	1.395 (4)
			
O4—Co1—O4^i^	180.00 (14)	H3A—C3—H3B	109.3
O4—Co1—O2	87.71 (9)	O5—C4—C3	102.3 (2)
O4^i^—Co1—O2	92.29 (9)	O5—C4—C5	101.9 (2)
O4—Co1—O2^i^	92.29 (9)	C3—C4—C5	111.7 (3)
O4^i^—Co1—O2^i^	87.71 (9)	O5—C4—H4A	113.3
O2—Co1—O2^i^	180.00 (6)	C3—C4—H4A	113.3
O4—Co1—O5^i^	92.19 (8)	C5—C4—H4A	113.3
O4^i^—Co1—O5^i^	87.81 (8)	C8—C5—C4	110.4 (2)
O2—Co1—O5^i^	90.69 (8)	C8—C5—C6	115.9 (2)
O2^i^—Co1—O5^i^	89.31 (8)	C4—C5—C6	100.7 (2)
O4—Co1—O5	87.81 (8)	C8—C5—H5A	109.8
O4^i^—Co1—O5	92.19 (8)	C4—C5—H5A	109.8
O2—Co1—O5	89.31 (8)	C6—C5—H5A	109.8
O2^i^—Co1—O5	90.69 (8)	C7—C6—C1	113.9 (2)
O5^i^—Co1—O5	180.00 (12)	C7—C6—C5	112.7 (2)
C15—S1—C14	90.24 (14)	C1—C6—C5	101.1 (2)
C15—N1—C13	114.3 (2)	C7—C6—H6A	109.6
C15—N1—H1N	121 (2)	C1—C6—H6A	109.6
C13—N1—H1N	125 (2)	C5—C6—H6A	109.6
C15—N2—H2C	120.0	O1—C7—O2	124.1 (3)
C15—N2—H2D	120.0	O1—C7—C6	118.3 (3)
H2C—N2—H2D	120.0	O2—C7—C6	117.7 (3)
H1WA—O1W—H1WB	104 (3)	O3—C8—O4	123.0 (3)
C7—O2—Co1	117.83 (18)	O3—C8—C5	118.9 (3)
H2WA—O2W—H2WB	104 (3)	O4—C8—C5	118.0 (3)
H3WA—O3W—H3WB	104 (3)	C14—C9—C10	118.4 (3)
C8—O4—Co1	127.4 (2)	C14—C9—H9A	120.8
C4—O5—C1	95.5 (2)	C10—C9—H9A	120.8
C4—O5—Co1	117.10 (17)	C9—C10—C11	120.3 (3)
C1—O5—Co1	111.96 (16)	C9—C10—H10A	119.8
O5—C1—C2	101.5 (2)	C11—C10—H10A	119.8
O5—C1—C6	102.2 (2)	C10—C11—C12	121.7 (3)
C2—C1—C6	111.5 (3)	C10—C11—H11A	119.1
O5—C1—H1A	113.5	C12—C11—H11A	119.1
C2—C1—H1A	113.5	C13—C12—C11	117.7 (3)
C6—C1—H1A	113.5	C13—C12—H12A	121.1
C1—C2—C3	102.2 (3)	C11—C12—H12A	121.1
C1—C2—H2A	111.3	C12—C13—N1	126.7 (3)
C3—C2—H2A	111.3	C12—C13—C14	121.1 (3)
C1—C2—H2B	111.3	N1—C13—C14	112.2 (3)
C3—C2—H2B	111.3	C9—C14—C13	120.7 (3)
H2A—C2—H2B	109.2	C9—C14—S1	128.9 (3)
C4—C3—C2	101.5 (2)	C13—C14—S1	110.4 (2)
C4—C3—H3A	111.5	N2—C15—N1	123.8 (3)
C2—C3—H3A	111.5	N2—C15—S1	123.3 (2)
C4—C3—H3B	111.5	N1—C15—S1	112.9 (2)
C2—C3—H3B	111.5		
			
O4—Co1—O2—C7	−42.6 (2)	C2—C1—C6—C5	72.9 (3)
O4^i^—Co1—O2—C7	137.4 (2)	C8—C5—C6—C7	−3.8 (3)
O5^i^—Co1—O2—C7	−134.8 (2)	C4—C5—C6—C7	−122.9 (3)
O5—Co1—O2—C7	45.2 (2)	C8—C5—C6—C1	118.2 (3)
O2—Co1—O4—C8	58.0 (2)	C4—C5—C6—C1	−0.9 (3)
O2^i^—Co1—O4—C8	−122.0 (2)	Co1—O2—C7—O1	139.8 (2)
O5^i^—Co1—O4—C8	148.6 (2)	Co1—O2—C7—C6	−40.9 (3)
O5—Co1—O4—C8	−31.4 (2)	C1—C6—C7—O1	152.8 (3)
O4—Co1—O5—C4	−10.50 (18)	C5—C6—C7—O1	−92.7 (3)
O4^i^—Co1—O5—C4	169.50 (18)	C1—C6—C7—O2	−26.5 (4)
O2—Co1—O5—C4	−98.23 (18)	C5—C6—C7—O2	87.9 (3)
O2^i^—Co1—O5—C4	81.77 (18)	Co1—O4—C8—O3	−167.9 (2)
O4—Co1—O5—C1	98.29 (18)	Co1—O4—C8—C5	16.1 (4)
O4^i^—Co1—O5—C1	−81.71 (18)	C4—C5—C8—O3	−128.2 (3)
O2—Co1—O5—C1	10.55 (18)	C6—C5—C8—O3	118.2 (3)
O2^i^—Co1—O5—C1	−169.45 (18)	C4—C5—C8—O4	48.0 (3)
C4—O5—C1—C2	−57.1 (3)	C6—C5—C8—O4	−65.7 (3)
Co1—O5—C1—C2	−179.28 (17)	C14—C9—C10—C11	−0.3 (5)
C4—O5—C1—C6	58.2 (2)	C9—C10—C11—C12	0.4 (5)
Co1—O5—C1—C6	−64.0 (2)	C10—C11—C12—C13	−0.5 (5)
O5—C1—C2—C3	35.8 (3)	C11—C12—C13—N1	−179.8 (3)
C6—C1—C2—C3	−72.4 (3)	C11—C12—C13—C14	0.5 (5)
C1—C2—C3—C4	−0.9 (3)	C15—N1—C13—C12	179.5 (3)
C1—O5—C4—C3	56.9 (3)	C15—N1—C13—C14	−0.8 (4)
Co1—O5—C4—C3	175.04 (17)	C10—C9—C14—C13	0.4 (5)
C1—O5—C4—C5	−58.7 (3)	C10—C9—C14—S1	180.0 (3)
Co1—O5—C4—C5	59.4 (2)	C12—C13—C14—C9	−0.5 (5)
C2—C3—C4—O5	−34.6 (3)	N1—C13—C14—C9	179.7 (3)
C2—C3—C4—C5	73.7 (3)	C12—C13—C14—S1	179.8 (2)
O5—C4—C5—C8	−86.5 (3)	N1—C13—C14—S1	0.1 (3)
C3—C4—C5—C8	165.0 (3)	C15—S1—C14—C9	−179.2 (3)
O5—C4—C5—C6	36.5 (3)	C15—S1—C14—C13	0.5 (2)
C3—C4—C5—C6	−72.0 (3)	C13—N1—C15—N2	−179.9 (3)
O5—C1—C6—C7	86.2 (3)	C13—N1—C15—S1	1.2 (3)
C2—C1—C6—C7	−166.0 (3)	C14—S1—C15—N2	−179.9 (3)
O5—C1—C6—C5	−34.9 (3)	C14—S1—C15—N1	−0.9 (2)

Symmetry codes: (i) −*x*+1, −*y*, −*z*.

### Hydrogen-bond geometry (Å, °)

**Table:**

*D*—H···*A*	*D*—H	H···*A*	*D*···*A*	*D*—H···*A*
N1—H1N···O1^ii^	0.84 (2)	1.85 (2)	2.675 (3)	169 (3)
N2—H2C···O2^ii^	0.86	2.00	2.851 (3)	173
N2—H2D···O2W^iii^	0.86	2.01	2.828 (4)	160
O1W—H1WA···O3W^iii^	0.82 (2)	2.21 (2)	3.030 (4)	176 (4)
O1W—H1WB···O3W^iv^	0.84 (4)	1.94 (2)	2.769 (4)	171 (5)
O2W—H2WA···O3	0.85 (2)	1.85 (2)	2.686 (3)	167 (4)
O2W—H2WB···O1W	0.83 (2)	1.95 (2)	2.772 (4)	171 (4)
O3W—H3WA···O1	0.84 (2)	2.01 (2)	2.815 (3)	160 (4)
O3W—H3WB···O2W	0.83 (4)	1.96 (4)	2.790 (4)	178 (4)

Symmetry codes: (ii) −*x*+1, −*y*, −*z*+1; (iii) −*x*+1, −*y*+1, −*z*+1; (iv) *x*−1, *y*, *z*.
